# Tachy-Brady Syndrome in a Schizophrenic Patient: A Case Report

**DOI:** 10.7759/cureus.35557

**Published:** 2023-02-27

**Authors:** Aaron D Knight, Kimberly Medina-Morales, Saleha Ozair, Sabas Gomez

**Affiliations:** 1 Internal Medicine, Larkin Community Hospital, South Miami, USA; 2 Internal Medicine, Medical University of the Americas, Miami, USA; 3 Cardiology, Larkin Community Hospital, South Miami, USA

**Keywords:** mental health issues, paranoid schizophrenia, dual-chamber pacemaker, metoprolol, ventricular arrhythmia, atrial arrhythmia, adult cardiology, sick sinus, tachy-brady syndrome

## Abstract

Tachy-brady syndrome is the result of sinus node dysfunction (SND), an electrocardiographic phenomenon caused by defective pacemaker functioning that leads to alternating arrhythmias. We present a case of a 73-year-old male with multiple mental health and medical comorbidities who was admitted to the inpatient floor for catatonia, paranoid delusions, refusal to eat, inability to cooperate with activities of daily life, and generalized weakness. Upon admission, a 12-lead electrocardiogram (ECG) showed an episode of atrial fibrillation with a ventricular rate of 64 beats per minute (bpm). During hospitalization, telemetry recorded a variety of arrhythmias such as ventricular bigeminy, atrial fibrillation, supraventricular tachycardia (SVT), multifocal atrial contractions, and sinus bradycardia. Each episode spontaneously reverted and the patient remained asymptomatic throughout these arrhythmic changes. These frequently fluctuating arrhythmias on resting ECG confirmed the diagnosis of tachycardia-bradycardia syndrome, also known as tachy-brady syndrome. Medical intervention, especially for cardiac arrhythmias, in patients with paranoid and catatonic schizophrenia can be challenging, as they might not share their symptoms. Additionally, certain psychotropic medications can also cause cardiac arrhythmias and must be carefully evaluated. The decision was made to start the patient on a beta-blocker and direct oral anticoagulation for reducing the risk of thromboembolic events. Due to an unsatisfactory response to drug therapy alone, the patient qualified as a candidate for definitive treatment with an implantable dual-chamber pacemaker. Our patient had a dual-chamber pacemaker implanted to prevent bradyarrhythmias and continued oral beta-blockers to prevent tachyarrhythmias.

## Introduction

Tachycardia-bradycardia syndrome, also known as tachy-brady syndrome, is the result of sinus node dysfunction (SND), which is characterized as an electrocardiographic phenomenon caused by defective pacemaker functioning that leads to alternating arrhythmias [1]. In a healthy heart, the sinus node continuously generates electrical impulses that are then transmitted throughout the heart thus setting the rate and rhythm of cardiac contractions [2]. However, dysfunction of the sinus node leads to abnormal conduction within the atrial tissue which can manifest as sinus bradycardia, sinus arrest, sinus exit block, atrial fibrillation, supraventricular tachycardia (SVT), or tachy-brady syndrome [1]. Although sinus node dysfunction is primarily related to degenerative fibrosis of nodal tissue in the elderly, it may occur at any age secondary to other cardiac abnormalities or extrinsic causes [2]. Some psychotropic medications, which our patient was taking, can also cause cardiac arrhythmias adversely [2]. Our patient's medications were carefully evaluated but ultimately excluded as the cause of these arrhythmic episodes. Given the intermittent and dynamic nature of these arrhythmias, patients can remain asymptomatic, which poses diagnostic challenges [2]. Additionally, diagnosing and treating patients with arrhythmias with underlining mental health conditions like schizophrenia is especially difficult. Here we present a case of tachycardia-bradycardia syndrome that initially presented as SVT but was later diagnosed to be SND and eventually needed an implantable pacemaker.

## Case presentation

A 73-year-old male (height: 165 cm, weight: 60 kg) with a past medical history of schizophrenia, anxiety, major depressive disorder (MDD), chronic obstructive pulmonary disease (COPD), gastroesophageal reflux disease, and constipation presented from an assisted living facility. This patient was admitted to the inpatient hospital for catatonia, paranoid delusions, refusal to eat, inability to cooperate with activities of daily life, and generalized weakness. His home medications included haloperidol, divalproex, lorazepam, citalopram, levalbuterol inhaler, ipratropium inhaler, pantoprazole, and docusate sodium. Social history was significant for smoking (27 pack-years) but negative for alcohol and illicit drug use.

**Figure 1:**
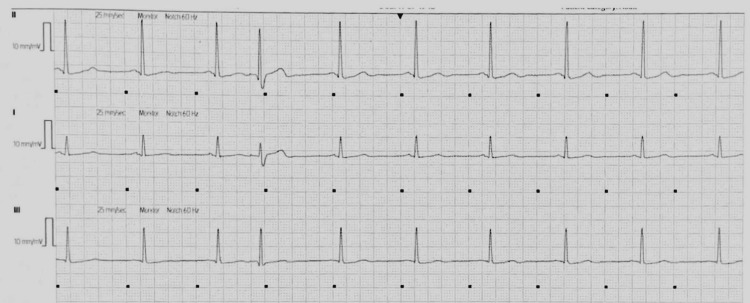
Patient's baseline rhythm recorded on telemetry - sinus rhythm with premature contraction Seen on Day 1

Upon admission to the hospital, his blood pressure was 132/86 mmHg, heart rate of 72 bpm, respiratory rate of 16 breaths per minute, and oxygen saturation of 98%. His ECG showed an episode of atrial fibrillation with a ventricular rate of 64 bpm lasting less than 5 minutes, which spontaneously resolved to a normal sinus rhythm at 84 bpm. The patient was subsequently placed on continuous monitoring via cardiac telemetry during his hospitalization. Over the course of the first night of hospitalization, telemetry recorded varying arrhythmias, including ventricular bigeminy at a rate of 74 bpm, atrial fibrillation at 88 bpm, and supraventricular tachycardia (SVT) at 206 bpm. Each arrhythmia lasted less than 5 minutes in duration and spontaneously reverted to a sinus rhythm of 82 to 96 bpm with frequent premature atrial or ventricular contractions, which was thought to be his baseline rhythm. Figure 1 is an ECG strip of the patient's baseline rhythm. The patient continued to remain asymptomatic throughout these arrhythmic changes. 

**Figure 2:**
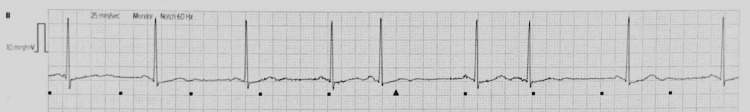
Sinus bradycardia at 47 beats per minute with premature contractions recorded on telemetry Seen on Day 2

**Figure 3:**
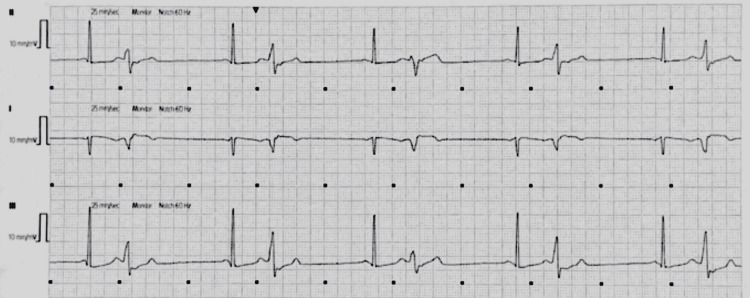
Ventricular bigeminy of 52 beats per minute recorded on telemetry Seen on Day 2

On the second hospitalized day, the patient’s rhythm changed twice from baseline, sinus bradycardia at 47 bpm and ventricular bigeminy at 52 bpm, before reverting to his baseline (sinus rhythm with premature contractions). Figure 2 shows an ECG strip of the sinus bradycardia, while Figure 3 shows an ECG of the ventricular bigeminy.

**Figure 4:**
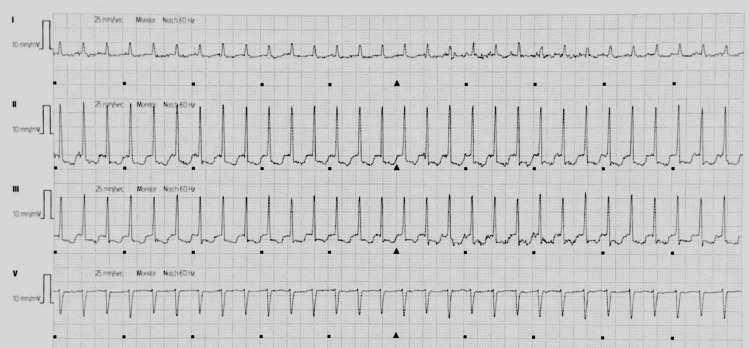
Supraventricular tachycardia at 178 beats per minute recorded on telemetry Seen on Day 3

On the patient’s third day of hospitalization, he experienced an episode of atrial fibrillation with a rapid ventricular response of 111 bpm and SVT at 178 bpm, again with each intervaled arrhythmia lasting less than 5 minutes, before returning to his baseline of sinus rhythm with frequent premature contractions. Figure 4 depicts an ECG strip of this witnessed SVT.

On day 4, the patient again had a change in his cardiac rhythm, multifocal atrial contractions at 70 bpm, and two episodes of SVT (172 bpm and 211 bpm), before reverting to his baseline sinus rhythm. These frequently fluctuating arrhythmias on resting ECG confirmed the diagnosis of tachy-brady syndrome and the patient was started on metoprolol tartrate 25 mg PO Q12h for rate control. The patient had a CHA₂DS₂-VASc score of 1, and a HAS-BLED score of 1 but apixaban 5 mg PO BID was started owing to the high risk of stroke, bleeding, and thromboembolic events in tachy-brady syndrome [3].

Due to the patient's extensive mental health conditions and state, the psychiatry team evaluated the patient. It was determined that the patient did not have the capacity to make major medical decisions but was capable to refuse his daily medical treatment and physical exams. Since this patient had paranoid delusions, treating him pharmacologically with beta-blockers and oral anticoagulants was extremely challenging. On some days he would allow medication administration, but on other days he would refuse. Furthermore, it was difficult to assess any cardiac symptoms during arrhythmic changes since he was slightly catatonic and rarely complained. Thus, he remained asymptomatic.

Over the course of the following four days, the patient fluctuated between sinus bradycardia to supraventricular tachycardia despite the initiation of beta-blocker therapy. However, the patient continued to remain asymptomatic and hemodynamically stable throughout these episodes. Table 1 further itemizes and describes each recorded arrhythmic fluctuation during his hospitalization.

**Table 1:** Fluctuating arrhythmic changes from patient’s baseline *Baseline Rhythm from Days 1-17; bpm: beats per minute; SVT: supraventricular tachycardia; PACs: premature atrial contractions

Day	Arrhythmia	Rate
*Baseline Rhythm	Sinus Rhythm with Premature Atrial/Ventricular Contractions	65-98 bpm
Day 1	Atrial Fibrillation	64 bpm
	Ventricular Bigeminy	52 bpm
	Atrial Fibrillation	88 bpm
	SVT	206 bpm
Day 2	Sinus Bradycardia with Premature Contractions	47 bpm
	Ventricular Bigeminy	74 bpm
Day 3	Atrial Fibrillation	111 bpm
	SVT	178 bpm
Day 4	Multifocal Atrial Contractions	70 bpm
	SVT	172 bpm
	SVT	211 bpm
Day 5	SVT	188 bpm
	Sinus Bradycardia	52 bpm
Day 6	Sinus Bradycardia with PACs	57 bpm
	SVT	190 bpm
Day 7	Sinus Bradycardia	47 bpm
Day 8	Sinus Bradycardia	50 bpm
	SVT	214 bpm
Day 9	SVT	178 bpm
	Atrial Fibrillation	82 bpm
Day 10	Sinus Bradycardia	47 bpm
Day 11	Sinus Bradycardia	50 bpm
	SVT	180 bpm
	Ventricular Bigeminy	88 bpm
Day 12	SVT	174 bpm
	Sinus Bradycardia	50 bpm
	Ventricular Bigeminy	67 bpm
Day 13	Sinus Bradycardia with PACs	47 bpm
Day 14	Sinus Bradycardia	50 bpm
	Atrial Fibrillation	63 bpm
Day 15	Sinus Bradycardia	47 bpm
Day 16	Ventricular Bigeminy	70 bpm
Day 17	Sinus Bradycardia	47 bpm
	Ventricular Bigeminy	74 bpm

Due to an unsatisfactory response to drug therapy alone, the patient qualified as a candidate for definitive treatment with an implantable dual-chamber pacemaker. The patient refused surgical intervention, but his healthcare proxy was in favor of the pacemaker placement. Based on his lack of capacity for major medical decisions, his medical proxy consented and the dual-chamber pacemaker was successfully implanted. By combining the pacemaker and metoprolol therapy, the alternations of bradycardia and tachycardia in this patient were successfully controlled and the patient was discharged back to the assisted living facility in a stable condition. Upon discharge, the patient was instructed to resume his home medications, as well as continue taking metoprolol tartrate 25 mg PO BID and apixaban 5 mg PO BID.

## Discussion

One in 600 cardiac patients over the age of 65 has sick sinus syndrome with an average age of 74 years [1]. Tachy-brady syndrome is a disorder caused by the dysfunction of the sinus node resulting in the inability to maintain its pacemaker function and impulse transmission throughout the cardiac conduction system [2]. Tachy-brady syndrome encompasses a myriad of variable arrhythmias that could be seen on an electrocardiogram, including sinus bradycardia, sinus arrest, sinus exit block, and tachycardia-bradycardia syndrome (tachy-brady syndrome) [4]. Tachy-brady syndrome is characterized by alternating patterns of bradycardia and tachycardia [4]. Although a patient with sinus node dysfunction could display a variety of arrhythmias, the alternating fluctuations of fast and slow heart rates, which define tachy-brady syndrome, are seen in approximately 50% of all sick sinus cases [2, 4].

The etiological factors can be associated with the degeneration of the sinus node and are classified as either intrinsic or extrinsic [2]. Intrinsic factors include infiltrative diseases, age-related degenerative fibrosis, ion channel dysfunction, and remodeling of the sinoatrial (SA) node [2]. Extrinsic factors include autonomic dysfunction, vagal tone abnormalities, metabolic disturbances, obstructive sleep apnea, medication adverse effects, and toxins [2]. While these factors all play a role in the development of sick sinus syndrome, current evidence shows that a combination of fibrosis and ion channel dysfunction is the cause of tachy-brady syndrome [5]. SA nodal fibrosis causes downregulation of a specific type of ion channel called hyperpolarization-activated, cyclic nucleotide-gated 4 (HCN4) channels [5]. HCN4 channels are types of sodium and potassium channels that are pivotal in the generation of an electrical impulse [5]. Dysfunction of the HCN4 channels leads to adenosine release, promoting bradyarrhythmias [5]. Additionally, SA node fibrosis and HCN4 channel dysfunction impair the electrical conduction throughout the cardiac system, which gives rise to re-entry circuits [5]. These re-entry circuits propagate tachyarrhythmias. Thus, fibrosis and ion channel dysfunction play a significant role in the pathogenesis of alternating tachycardic and bradycardic rhythms in sinus node dysfunction [2, 5].

The majority of excess mortality among people with schizophrenia seems to be caused by cardiovascular complications, particularly coronary heart disease [6]. In addition, the prevalence of heart failure and arrhythmias has increased in the schizophrenic population [6]. Although psychotropic medications play a key role in the management of schizophrenia and MDD, these drugs have significant cardiac effects. Haloperidol is considered minimally cardiotoxic, but this medication can cause Torsade de Pointes, or polymorphic ventricular tachycardia [7]. Although, the Torsade de Pointes is typically preceded by QTc prolongation, which is used as a predicting factor for ventricular arrhythmias [7]. Divalproex, which works through neuronal sodium channel blockage, can also cause cardiac sodium channel blockade [8]. Through inhibition of the cardiac sodium channel, divalproex can cause clinically significant effects on cardiac electrophysiology, leading to cardiac arrhythmias [8]. Lorazepam can cause a dose-dependent increase in cardiac vagal tone, resulting in a decrease in heart rate [9]. This decreased heart rate could potentially cause bradycardia, although lorazepam has a minimal effect on blood pressure [9]. Among selective serotonin reuptake inhibitors (SSRIs), citalopram is known to have the highest cardiotoxic capacity in a dose-dependent fashion [10]. A wide range of conduction disturbances and arrhythmias have been reported vis-a-vis citalopram intake, including sinus bradycardia and tachycardia, left and right bundle branch block, supraventricular tachycardia, ventricular fibrillation, and QTc prolongation [10, 11]. Therefore, clinicians should carefully monitor for these adverse effects [10, 11]. Nevertheless, this patient continued to remain asymptomatic and hemodynamically stable throughout these arrhythmic episodes, which makes a medication-adverse effect not likely for his diagnosis.

While pharmacologic agents, like beta-blockers and non-dihydropyridine calcium-channel blockers, could treat tachyarrhythmias, these drugs could actually worsen the fluctuating bradyarrhythmias [12]. Implanted permanent pacemakers could prevent bradyarrhythmias from forming, but they will not stop the heart from going into a tachyarrhythmia [4]. Hence, the proper treatment for tachycardia-bradycardia syndrome is simultaneously utilizing a permanent pacemaker and administering medications like beta-blockers or calcium-channel blockers [4, 5, 12].

Our patient had a dual-chamber pacemaker implanted to prevent bradyarrhythmias and was given oral metoprolol to prevent tachyarrhythmias [13]. A dual-chamber pacemaker paces both the right atria and right ventricle, which optimizes ventricular filling and cardiac output by maintaining a normal atrial-ventricular sequence [13]. For patients with tachy-brady syndrome, the major benefit of a dual-chamber pacemaker compared to a standard pacemaker, which only paces the right ventricle, is the prevention of atrial fibrillation [13]. By minimizing the possibility of atrial fibrillation, the chance of clot formation along the wall of the poorly contracting atria is also decreased [13]. However, since the risk of atrial fibrillation is not completely reduced, oral apixaban was given to our patient to further decrease the chance of mural thrombus formation, systemic thromboembolism, stroke, and death [3].

Oral metoprolol, a cardio-selective (beta-1) beta-blocker, is considered a first-line pharmacologic agent in maintaining ventricular rate control, and was, therefore, added [14]. The Mode Selection clinical trial (MOST) concluded that dual-chamber pacing does not improve stroke-free survival, but it does reduce the risk of atrial fibrillation, reduces signs and symptoms of heart failure, and improves the overall quality of life [15]. Data suggests that establishing a temporal correlation between symptoms and episodes of bradycardia is important when assessing the need for a permanent pacemaker [13, 16]. Per 2018 American College of Cardiology (ACC) guidelines, there is no established minimum heart rate** **or pause duration where permanent pacing is recommended in sick sinus syndrome [16].

## Conclusions

Tachycardia-bradycardia syndrome, also known as tachy-brady syndrome, is the result of sinus node dysfunction (SND), which is characterized as an electrocardiographic phenomenon caused by defective pacemaker functioning that leads to alternating arrhythmias. Beta-blockers and non-dihydropyridine calcium-channel blockers are used to treat tachyarrhythmias with the addition of an implantable permanent pacemaker to prevent bradyarrhythmias. Medical intervention and treatment for patients with mental health issues like schizophrenia can be challenging. Cardiac arrhythmias can be difficult to manage, especially in schizophrenic patients with catatonic and paranoid behaviors, as they might not share their symptoms. Additionally, psychotropic medications can cause cardiac abnormalities on ECG. Clinicians should have a low threshold for including tachy-brady syndrome as part of their differential diagnoses for all patients presenting with tachyarrhythmias, regardless of their symptoms. Early diagnosis can lead to a timely approach to definitive management and better outcomes.
